# Antimicrobial Polymer Surfaces Containing Quaternary Ammonium Centers (QACs): Synthesis and Mechanism of Action

**DOI:** 10.3390/ijms25147587

**Published:** 2024-07-10

**Authors:** Orlando Santoro, Lorella Izzo

**Affiliations:** Department of Biotechnology and Life Sciences, University of Insubria, 21100 Varese, Italy; orlando.santoro@uninsubria.it

**Keywords:** antimicrobial surfaces, antimicrobial polymers, quaternary ammonium centers, antimicrobial mechanism

## Abstract

Synthetic polymer surfaces provide an excellent opportunity for developing materials with inherent antimicrobial and/or biocidal activity, therefore representing an answer to the increasing demand for antimicrobial active medical devices. So far, biologists and material scientists have identified a few features of bacterial cells that can be strategically exploited to make polymers inherently antimicrobial. One of these is represented by the introduction of cationic charges that act by killing or deactivating bacteria by interaction with the negatively charged parts of their cell envelope (lipopolysaccharides, peptidoglycan, and membrane lipids). Among the possible cationic functionalities, the antimicrobial activity of polymers with quaternary ammonium centers (QACs) has been widely used for both soluble macromolecules and non-soluble materials. Unfortunately, most information is still unknown on the biological mechanism of action of QACs, a fundamental requirement for designing polymers with higher antimicrobial efficiency and possibly very low toxicity. This mini-review focuses on surfaces based on synthetic polymers with inherently antimicrobial activity due to QACs. It will discuss their synthesis, their antimicrobial activity, and studies carried out so far on their mechanism of action.

## 1. Introduction

One of the routes for pathogen dissemination is via contaminated surfaces, as most bacteria can survive for a long time even on the surface of objects. Nosocomial infections generated by contaminated surfaces are a great concern all over the world. Once the human body and a medical device or implant come into contact, a door opens for bacteria to enter. For example, patients with a urinary catheter present an infection risk of 50% after 10 days, and 100% after 30 days [1]. However, common disinfectants used in routine cleansing, generally based on quaternary ammonium compounds, halogen releasing agents, and phenolics, are in some cases ineffective at killing pathogenic bacteria, as their activity depends on several factors, including the surface contact period, pH, temperature, and amount and nature of the microorganisms [2]. In the past two decades, to obtain decontamination systems, different antimicrobial substances have been incorporated in the bulk or as a coating of surfaces. They can be added during the phase of production, a posteriori absorbed, or covalently bound to functionalized materials, e.g., polymers [3,4]. The storage of anti-pathogens in bulk materials is one of the approaches used for gradually releasing biocides, which provides sustained delivery able to kill pathogens over time. However, at least three disadvantages can be glimpsed in such an approach: (i) an excessive use of antibiotics that can lead to the development of antibiotic resistance; (ii) the end of antimicrobial activity once the leaching component is exhausted, which may foster bacterial resistance when the drug doses become sub-lethal; and (iii) the environmental contamination and accumulation of non-degradable leaching components that may generate resistance in environmental bacteria [5]. To this end, it is worth noting that over the last few decades, leading health organizations have warned about the globally increasing numbers of antibiotic-resistant bacteria among pathogens, in both hospitals and communities [6,7,8,9].

Non-releasing polymeric surfaces allow for the overcoming of issues related to the use of releasing systems, provided that the polymer is inherently antimicrobial.

**Figure 1:**
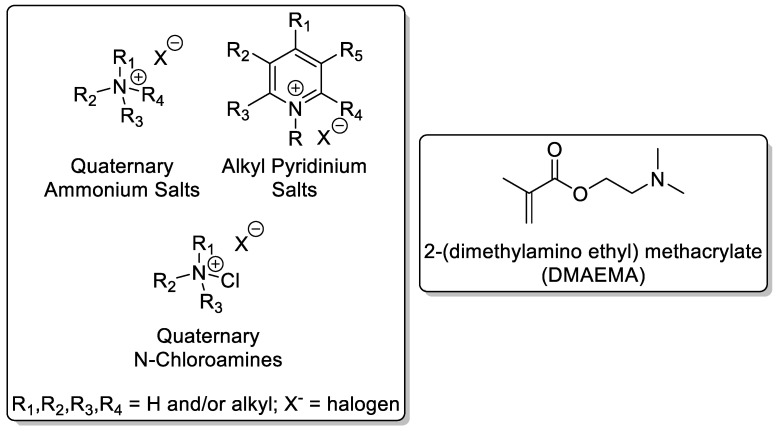
General formulas of quaternary ammonium centers (QACs) and structure of 2-(dimethylamino ethyl) methacrylate (DMAEMA).

Most of the surface-forming polymers are cationic and contact-killing and, most importantly, they do not induce serious microbial drug resistance as they produce physical damage to bacterial cells or exert non-specific oxidative stress instead of addressing specific targets such as ribosomes [10], while the presence of cationic moieties facilitates the interaction with the surface of the microbial membrane. Among the cationic groups, the use of quaternary ammonium centers (QACs) as antiseptics and disinfectants dates to the 1930s [11]. Structurally, QACs are positively charged organic molecules containing hydrogens and/or alkyl groups covalently attached to a central nitrogen atom. QACs may be generated by protonation of primary, secondary, or tertiary amine groups, so that the extent of the quaternarization is pH-dependent (according to the pK_b_ of the amine) or they possess a permanent charge deriving from the nitrogen attached to four groups by covalent bonds such as in alkyl pyridiniums, quaternized amines, and N-chloroamines (Figure 1). Polymers bearing primary, secondary, and tertiary protonated amines usually have low hemolytic activity compared with those containing QACs with a permanent charge [12,13].

In this contribution, recent developments in the field of antimicrobial polymer surfaces are reviewed, with a particular focus on those containing protonated or quaternized amines and alkyl pyridinium as QACs. Several important aspects will be addressed, such as the synthesis of the most used QAC-based materials, in terms of both coated surfaces and intrinsically antimicrobial surfaces, and the proposed mechanism of action depending on the QAC-containing polymer structures.

## 2. Antimicrobial Polymer Surfaces Containing QA Moieties

**Scheme 1:**
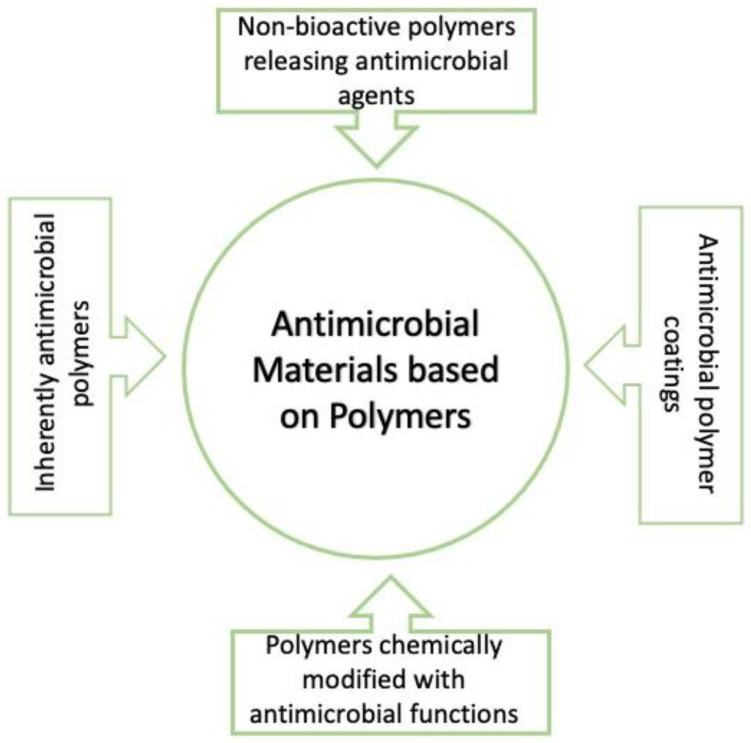
Representation of possible antimicrobial systems based on polymers.

A large variety of polymers and copolymers have been studied with the aim of enhancing their antimicrobial activity by the density of charges, hydrophobicity, molecular weights, and other parameters, and several techniques have been used to create polymeric antimicrobial surfaces. A few of such surfaces consist of a coating exhibiting organic or inorganic substrates with antimicrobial polymers. Others are related to the formation of films deriving from thermoplastic antimicrobial polymers that are non-soluble in water (Scheme 1) [14].

In both cases, most of the surfaces that use QA moieties as antimicrobial groups are acrylic- or methacrylic-based polymers, and generally contain 2-(dimethylamino ethyl) methacrylate (DMAEMA) as the monomer (Figure 1).

In this review, we discuss the formation of antimicrobial polymer surfaces, considering two categories: polymer coatings generated by covalently immobilizing polymer chains to existing surfaces, and the formation of films from inherently antimicrobial polymers.

### 2.1. Antimicrobial Polymer Coating Covalently Attached to Surfaces

**Scheme 2:**
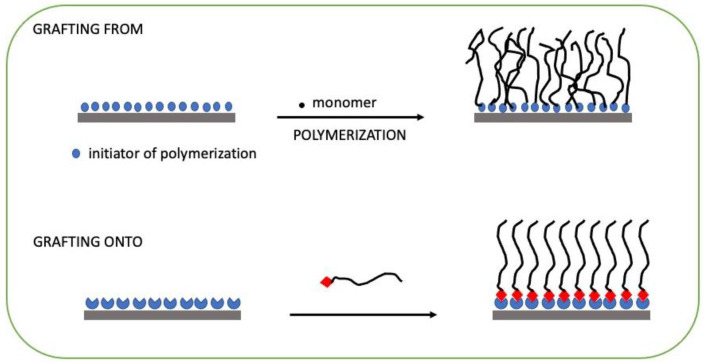
Schematic representation of the “grafting from” and the “grafting onto” techniques.

Along with the possibility of coating surfaces from either organic solvents or aqueous solutions, to produce an antimicrobial polymer-based coating on existing surfaces, either the “grafting from” or the “grafting onto” technique may be used, both displaying advantages and disadvantages (Scheme 2) [15].

Generally, the “grafting onto” method is preferred when the surface already possesses functional groups able to anchor the preformed polymers, or when polymers with different architectures or chemical compositions need to be attached to the surface. However, surfaces with high grafting density are not generally obtained, due to the increasing steric hindrance of the chains that are gradually attached to the surface. As a matter of fact, the mushroom-like structure formed after the reaction of the first polymers renders less accessible the remaining reactive sites on the surface to the incoming chains [16]. In addition, the electronic repulsion between the chains already containing QACs has a detrimental effect on the grafting efficiency. Finally, the “grafting onto” technique is not really applicable to high-molecular-weight polymers because of the scarce reactivity of their active termini with the sites on the surface [17]. On the other hand, the “grafting from” method is largely used to achieve a more uniform coating of the surface, and a good density and thickness of polymer distribution. However, it can be applied after a chemical modification of the surfaces to introduce functional groups acting as initiators of the polymerization processes.

Kugler and coworkers reported on the application of both methods to obtain glass surfaces grafted with quaternized poly(vinylpyridine) [18]. It was found that the charge density of the organic layer spanned from 10^12^ to 10^16^ charges/cm^2^, with the highest charge densities provided by the “grafting from” approach. The existence of a charge-density threshold depending on the bacterial strain and growth state (hence on their metabolism), and above which cell death occurs quickly upon adsorption on substrates, underline the importance of the method applied to obtain efficient antimicrobial surfaces.

Matyjaszewski et al. found as well that the “grafting from” approach produced surfaces containing quaternized DMAEMA with a higher charge density than the “grafting onto” technique (10^16^ charges/cm^2^ and 10^14^ charges/cm^2^, respectively) and consequently with a higher biocidal efficacy [19].

The “grafting from” approach is therefore largely used to obtain antimicrobial polymer-coated surfaces, and for this technique both free (FRP) and controlled radical polymerizations (CRP) are applied. Among the latter, atom transfer radical polymerization (ATRP) and its derived techniques have found large appeal in the synthesis of QAC-containing polymers on surfaces because of their tolerance towards monomers with polar functional groups and, in the case of its variant ARGET (Activators ReGenerated by Electron Transfer), also to adventitious oxygen present in the reaction systems [20]. This is due to the use of catalyst systems in which the metal is introduced in its higher (more stable) oxidation state and because of the presence of a reducing agent able to reactivate the catalyst after undesired termination processes. However, ATRP lets off large amounts of catalyst ashes that can compromise the safety of the final material when applied in biomedical fields.

In spite of this, the polymerization of DMAEMA via ATRP on filter papers followed by the quaternization of the amino groups, affording an antimicrobial material active against *Escherichia coli* and *Bacillus subtilis*, was reported by Matyjaszewski and coworkers. The antimicrobial activity was enhanced by increasing the density of polymer chains on the surface and consequently the QAC concentration [21]. After this seminal work, ATRP was used to polymerize DMAEMA on different surfaces such as silicon wafers [22,23], glass [19,24], stainless steel [25,26], Fe_3_O_4_ magnetite nanoparticles, silicon nanowire arrays [27], silicon catheters [28], and PVDF membranes [29].

**Figure 2:**
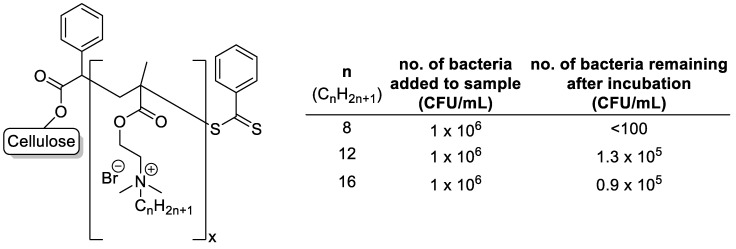
Relationship between antimicrobial activity against *E. coli* of quaternized PDMAEMA-coated cellulose and the chain length of the bromoalkene employed in the functionalization [30].

Another controlled radical technique used for the surface-initiated polymerization by the “grafting from” method is Reversible Addition Fragmentation chain Transfer (RAFT). RAFT can be easily used to polymerize both polar and nonpolar monomers under mild experimental conditions; in spite of the several advantages related to such a technique, it is somewhat limited by the choice of an appropriate chain transfer agent (CTA) specific for each monomer; indeed, in the absence of suitable commercially available CTAs, their design and synthesis are required. Roy and coworkers modified cellulose papers with PDMAEMA via RAFT, and quaternized the amino-pedant group of DMAEMA with alkyl bromides of different chain lengths (C_8_−C_16_) [30]. The antibacterial activity was affected both by the alkyl chain length and by the degree of quaternization: the higher the degree of quaternization and the shorter the alkyl chain, the higher the antimicrobial activity against *E. coli* (Figure 2).

RAFT was also applied to obtain a reversibly switchable bactericidal and antifouling surface combining the thermally responsive *N*-isopropylacrylamide (NIPAAm) and bactericidal quaternary ammonium salts of DMAEMA [31]. The copolymer coating was able to switch by phase transition between a hydrophobic capturing surface at 37 °C and a relatively hydrophilic antifouling surface at 4 °C. The bactericidal efficiency was proved against both *E. coli* and *S. aureus*. In particular, the bacteria were stained with live–dead two-color fluorescent dye, and the distribution of the dead/viable cells was investigated using fluorescence microscopy (FM) images. The presence of a large number of red-stained (dead) bacteria on the QAC-bearing material indicated the efficiency of the antimicrobial function; on the other hand, only living cells were observed on the negative control (glass) surface.

*Click chemistry*, a facile synthetic technique developed to covalently attach specific molecules to a substrate [32], has often been used in the “grafting onto” approach. Among the suitable organic reactions fulfilling such an approach, the copper-mediated cycloaddition of azides and alkynes (CuAAC) was used for the *click* grafting of azide-terminated PDMAEMA onto alkyne functionalized graphene oxide (GO-PDMAEMA). The attached PDMAEMA was then quaternized in the presence of ethyl bromide and the resulting functionalized graphene oxide showed excellent antibacterial activity, reducing nonspecific protein adsorption and cellular adhesion [33]. To avoid the use of potentially toxic metal catalysts, a Cu-free click chemistry approach [34] was adopted by Wang [35] to graft quaternized PDMAEMA (QPDMAEMA), modified with azide, to quantum dots (QDs), the latter derived from dibenzocyclooctynes (DBCOs). The QPDMAEMA-modified QDs were biocompatible and exhibited the ability of selective recognition and killing of bacterial cells of *E. coli* and *S. aureus*. The high negative charge density of the bacterial cell surface, in contrast with the near-neutrality of mammalian cells, represented the electrostatic driving force to bind selectively to bacteria but not mammalian cells for the positively charged QDs.

The thiol groups can react with C=C bonds through a *click chemistry* reaction under mild conditions, according to the thiol–ene Michael addition mechanism [36]. A quaternized dimer of DMAEMA, obtained by the addition of the tertiary amines to organo halides, was used by Tian et al. to make antimicrobic wool fabric via a thiol–ene reaction, involving the C=C bonds of the DMAEMA dimer and the thiol groups of the wool fabric after reduction of the disulfide bonds. The authors found that the antibacterial efficiencies of the modified wool fabric were 94.2% and 90.1% against *E. coli* and *S. aureus,* respectively. Furthermore, the thiol–ene reaction, having produced crosslinks for the presence of two reactive alkene groups for each DMAEMA dimer, increased the strength of the wool fabric [37].

Although both ATRP and RAFT polymerization techniques exhibit drawbacks (i.e., use of a metal catalyst that potentially leaches into the final material or weak bonds between the surface and the polymer), compared to other radical polymerization approaches, they allow for an even distribution of the polymer on the surface as well as for a higher control over the molecular weight and the polydispersity of the macromolecules, permitting a more homogeneous behavior of the biocidal materials, since all of the polymer chains exhibit similar natures [38].

### 2.2. Antimicrobial Surfaces Made of Inherently Antimicrobial Polymers

Compared to the plethora of soluble antimicrobial polymers containing QACs reported in the literature [39], examples of insoluble, self-consistent materials are scant. This could be due to the loss of antimicrobial activity sometimes observed when soluble polymers are post-functionalized (i.e., crosslinked), resulting in insoluble materials [40]. On the other hand, it has been shown that biocidal potential can still be preserved due to mechanisms that destabilize the bacterial membranes by contact (*vide infra*) [41].

Inherently antimicrobial polymers or copolymers can be classified as materials that (i) exhibit antimicrobial activity by themselves; (ii) are antimicrobial upon chemical modification; and (iii) incorporate antimicrobial organic compounds or active inorganic systems [42]. In this mini-review, we report on the first two classes of antimicrobial materials that form surfaces, meaning polymers that are not soluble in water.

One of the most common routes to obtain antimicrobial, non-soluble polymer surfaces consists in introducing antimicrobial functionalities in inactive polymers. This is because monomers containing QACs generally produce polymers soluble in water.

Punyani and coworkers reported a copolymer based on a quaternary amine methacrylate (QAMA) and 2-hydroxyethyl methacrylate (HEMA), produced by free radical bulk polymerization. The novel monomer QAMA was synthesized by amination of di(methacrylate) with piperazine followed by its quaternization with an alkyl iodide. The antibacterial activity against *Escherichia coli* and *Staphylococcus aureus* was studied by the zone of inhibition and colony count methods and the authors found that the QAMA copolymer showed broad-spectrum contact-killing antimicrobial properties without the release of any bioactive residue. Furthermore, the antimicrobial activity increased with increasing QAMA concentration in the copolymers [43]. QAMA was also used as a comonomer in copolymerization of methyl methacrylate (MMA) via partial substitution of the MMA monomer in commercial bone cements and by using the redox initiator activator system for curing. The presence of QAMA conferred antibacterial properties to the cements, while iodine imparted radiopacity. The antimicrobial tests evidenced no growth of *E. coli* onto the modified PMMA bone cement with 15% QAMA content, and a cytotoxicity test using a human cell model had a negative result [44,45]. Grafting polymerization of DMAEMA was used by Chung et al. [46] to confer hydrophilicity to the hydrophobic polyurethane (PU) surfaces. The grafted PDMAEMA affected PU properties such as thermal transition, shape memory, and flexibility at a very low temperature, while its hydrophilicity and its positive charge due to the protonated ammonium groups conferred antifungal effectiveness towards a mixture of fungi (*Aspergillus niger*, *Aureobasidium pullulans*, *Chaetomium globosum*, *Gliocladium virens*, and *Penicillium pinophilum*), provoking, in the case of the material prepared with 20 mol% of DMAEMA, the complete suppression of fungal growth.

Another polymer widely used in medical applications for the production of implants, namely polyether ether ketone (PEEK), has been modified by the self-initiated photoinduced graft polymerization of DMAEMA [47]. After grafting, the pedant amino groups of DMAEMA were quaternized using various bromoalkanes with different lengths of the alkyl chain (BrC_4_, BrC_8_, BrC_12_, and BrC_16_) to obtain Q-PDMA-*g*-PEEK [48]. A comparison between the relative antibacterial rates of the PDMA-g-PEEK and Q-PDMA-g-PEEK showed that the quaternization of the DMAEMA increased the antibacterial activity with the latter depending on the number of carbon atoms of the bromoalkanes. In particular, the antibacterial rate against *E. coli* of polymers quaternized with BrC_8_ was significantly different than that obtained using BrC_4_, BrC_12_, and BrC_16_. The authors concluded that the bactericidal properties of the materials were driven by their ζ-potential, a parameter that, along with the volume of the Q-PDMA layer grafted onto PEEK, is strictly related to the positive charge density of the surface; hence, the higher the ζ-potential, the greater the biocidal effect [49].

Poly(ethylene terephthalate) (PET) sheets were modified to introduce functions acting as the initiators for the ARGET-ATRP of DMAEMA. PET was treated first with dopamine, and then with 2-bromoisobutyryl bromide (BIBB). ARGET-ATRP was carried out using a catalyst system based on CuBr_2_ and *N*,*N*,*N*′,*N*″,*N*″-pentamethyl diethylenetriamine (PMDETA) and ascorbic acid as a reducing agent; finally, the terminal amino-pedant groups of the PDMAEMA-grafted PET sheets were quaternized by a reaction with 1,3-propiolactone and 1,3-propanesultone, affording polycarboxybetaine and polysulfobetaine brushes [50]. The authors demonstrated that with the increasing number of polyzwitterions, the *E. coli* cell adhesion decreased greatly. These results were mostly attributed to the antifouling property of polyzwitterions. Moreover, the cationic PDMAEMA chains and ammonium groups of polyzwitterions allegedly interacted with the negatively charged bacterial cell membrane, resulting in bacterial cytoplasmic membrane disruption and cell lysis [51]. The authors concluded that the synergistic weak positively charged and neutral zwitterionic surface would endow the surface with dual antifouling and antibacterial properties.

In 2006, Kenawy and coworkers reported on the synthesis via free radical polymerization of a series of crosslinked copolymers of vinylbenzyl chloride either with MMA or 2-chloroethylvinyl ether in the presence of divinylbenzene as the crosslinking agent [52]. Further reaction of such products with triethylamine afforded quaternized polymers displaying antimicrobial activity against fungi (*C. albicans* SC5314, *A. flavus*, and *F. oxysporum*) and bacteria (*B. subtilis*, *E. coli*, and *S. aureus).* Due to the insolubility of the materials, the antimicrobial potential was assessed by means of the cut plug method.

Biodegradable aliphatic polyesters displaying permanent biocidal activity have been recently described by Lecomte and coworkers [53]. The polymers were synthesized by tin-catalyzed ring opening copolymerization of ε-caprolactone and α-cloro-ε-caprolactone followed by the conversion of the pendant halides into azide groups. The latter functions were exploited in the Cu-catalyzed cycloaddition of *N*,*N*-dimethyl-*N*-prop-2-yn-1-yloctan-1-ammonium bromide for the introduction of the quaternized ammonium groups onto the polymer chain. These water-insoluble materials proved to be effective against *E. coli*, as assessed by the shake flask method. Interestingly, the parental ammonium salt, albeit soluble in the medium, displayed lower biocidal efficiency than the functionalized copolymers.

The bactericidal activity of films obtained from water-insoluble branched polymers containing non-quaternized DMAEMA was recently tested against *E. coli*, *S. aureus*, *Pseudomonas* sp., and *Dechlorosoma* sp. [54]. The materials were synthesized by the ATRP copolymerization of MMA and DMAEMA employing a polyethylene glycol-monomethylether (mPEG)-based macroinitiator. Such an approach afforded copolymers with architectures of the type A(BC)_n_, in which A and (BC) represented the mPEG and the MMA-*random*-DMAEMA blocks, respectively, and *n* the number of arms (1, 2, and 4). The structure of the copolymers proved to greatly influence their biocidal efficiency; indeed, for the gram-negative bacteria, the activity trend was found to be A(BC)_2_ ≥ A(BC)_4_ >> A(BC). The two-armed polymer also proved active against gram-positive species. The dependency of the antimicrobial activity on the polymer architecture was attributed to the formation of strong hydrogen bonds between proximal amino groups in the A(BC)_2_ films, while such interaction was rather disfavored in the case of the linear material.

Later, the antimicrobial activity of films made from branched A(BC)_n_ copolymers (n = 1, 2 or 4) bearing mPEG (A) and random copolymeric chains formed by MMA and *N*-alkyl aminoethyl methacrylates (AAEMAs) was studied [55]. *N*-alkyl substituents with progressively increasing hydrophobicity (Me, Et, *i*-Pr, *t*-Bu) were selected and series of copolymers with different AAEMA molar ratios (40 and 15%) were synthesized in order to vary, along with the molecular architecture, the hydrophobic/charge-density balance. For the polymers with a higher AAEMA fraction, the A(BC)_2_ structures displayed the highest charge density, and the DMAEMA-based material proved the most effective copolymer of the series against *E. coli*. On the other hand, the antimicrobial activity of the copolymer containing 15% of the AAEMA was found to be more dependent on the basicity of the pendant amino groups rather than on the molecular architecture; in fact, the DMAEMA-containing films exhibited lower antimicrobial activity with respect to those materials displaying amines with lower pK_b_ values.

Recently, the efficiency of copolymeric materials as additives for a commercial dental adhesive (AdheSE One F) conferring antimicrobial and collagenase-inhibiting properties was investigated by Tiller and coworkers [56]. The main backbone of the polymers was based on poly(2-methyloxazoline), while the terminals featured a polymerizable group (methacrylamide) on one side and a biocidal function (N-dodecyl-N,N-dimethylxylylammonium bromide) on the other. Upon employing 2.5 wt% of the additive, the material exhibited antimicrobial activity against *S. mutans* cells even after prolonged washing with water; in addition, higher concentrations (5 wt%) allowed for the killing of the bacteria remaining in the tubuli below the cured adhesive as well as for the complete inhibition of the human collagenase MMPs bound to dentin.

Copolymers of 3-(methacrylamidomethyl)-pyridine (MAMP) and *N*-isopropylacrylamide (NIPAAm) with different compositions, namely 50:50 and 90:10 MAMP:NIPAAm, were synthesized by free radical polymerization and further quaternized by a reaction of bromoalkanes with different chain lengths (C_12_, C_14_, and C_16_) [57]. While the water-soluble 90:10 composition copolymers exhibited antimicrobial activity against *E. coli* and *S. Aureus*, the insoluble materials obtained from the 50:50 analogues proved completely ineffective. In particular, the highest biocidal effect was exhibited by the 90/10 copolymer quaternized with the C_14_ bromoalkene, displaying Minimum Bactericidal Concentration (MBC) values of 320 and 160 μg/mL against *S. aureus* and *E. coli*, respectively, while the copolymers functionalized with C_12_ and C_16_ chains proved less active (MBCs of 640 and 320 μg/mL for *S. aureus* and *E. coli*, respectively).

The hydrophilicity of the polymers proved to have a profound effect on the bactericidal activity. Indeed, copolymers of 4-vinyl pyridine with either hydroxythylmethacrylate (HEMA) or polyethylene glycol methyl ether methacrylate (PEGMA), further quaternized with hexylbromide, proved to be ca. 20 times more active against *E. coli* than the parental quaternized homo poly(vinylpyridine) (PVP) [58]. This was attributed to the increased surface wettability of the copolymers containing a lower fraction of PVP.

Insoluble crosslinked poly-(vinylpyridium halide)-based resins were successfully employed in the removal of bacteria [59] and viruses [60,61] from water and air [62]. Nevertheless, in all cases the microorganisms were found to be only retained by the resins, which hence displayed poor biocidal activity. This was in stark contrast with the actual killing effect exhibited by previously reported tetraalkylammonium-type anion-exchange resins.

Cationic polymers containing positive nitrogen atoms in the main chain, namely ionene polymers, also possess antimicrobial properties due to the presence of QACs within the main chain [63]. In this *scenario*, polyelectrolytes obtained by condensation of benzyl amine and epichlorohydrin exhibited antimicrobial activity against bacteria (*P. aeruginosa*, *K. pneumoniae*, *E. feacalis*, *M. luteus*, and *B. subtilis* var. *niger*), yeast (*C. albicans*), and fungi (*P. digitatum* and *A. niger*) [64]. Interestingly, the extent of the biocidal potential was found to be dependent on the length of the polymer chains. An ionene-based, fast-swelling antimicrobial superabsorber able to kill the bacterial cells of the nosocomial strains *S. aureus*, *E. coli*, and *P. aeruginosa* has been recently reported by Tiller and coworkers [65].

Finally, in the field of modified polymers exhibiting antimicrobial activity, the use of polysaccharides has been very recently reviewed [66].

A summary of the contributions reviewed in this section is reported in Table 1.

## 3. Mechanism of Action

**Figure 3:**
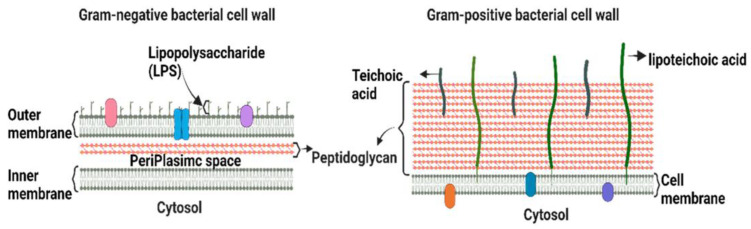
Schematic depiction of the key structural differences between gram-positive and gram-negative bacteria. Reproduced from reference [67].

Antimicrobial activity of polymers is influenced by multiple factors related to their physico-chemical properties, such as molecular mass, polymer charge, hydrophilicity, and external *stimuli* such as temperature and pH. Polymer surfaces, however, principally act via a contact-killing mechanism and consequently the charge density and the hydrophobicity of surfaces play a predominant role, while layer thickness and molecular weight might influence the activity when prompted by different surface charge densities. Nevertheless, the mechanism of the contact-killing action is still under debate, and it has to be mentioned that the biocidal release mechanism cannot be simply ruled out by inhibition zone tests. Indeed, the presence of an inhibition zone indicates a large extent of release without proving the mechanism. The occurrence of a contact-killing pathway can be confirmed upon assessing the biocidal efficiency of the material after long washing cycles. Polymer surfaces containing QACs are positively charged while, as is known, the external envelope of bacteria exhibits a net negative charge generally stabilized by divalent cations such as Ca^2+^ and Mg^2+^. The negative charge derives from the chemical composition of the external envelope in both gram-positive and -negative bacteria: the former possess teichoic or lipothecoic acids in the cell wall (CW), while lipopolysaccharides and phospholipids can be found in gram-negative bacteria’s outer membrane (OM). The cytoplasmic membrane (CM) is also negatively charged, being composed of phospholipids and embedded proteins (Figure 3) [67].

It is generally accepted that positively charged water-soluble antimicrobial polymers must possess some level of hydrophobicity to allow initial polymer adhesion to negatively charged cell surfaces and further membrane penetration and permeabilization, although biocidal hydrophilic polycations capable of penetrating bacterial cells without disrupting the membrane have been very recently described [68]. In 1986, Ikeda et al. [69] reported that the lysis of protoplasts of *Bacillus subtilis* showed that polymers containing QACs were able to damage the CM of cells with release of cytoplasmic constituents such as K^+^, DNA, and RNA. These results were confirmed by more recent studies carried out with techniques aiming at monitoring the loss of cell constituents and demonstrating that QACs of synthetic polymers provoked cell lysis and consequently cell death by damaging the OM as well as the CM [70,71,72,73,74,75,76].

A similar mechanism of action has been hypothesized for polymeric surfaces, even if the hydrophobic contribution is not clear in this case. Furthermore, with respect to soluble chains, the antimicrobial biocidal surfaces present the charged groups tethered to the surface, hence they cannot be released to completely penetrate the envelope to reach the CM; for this reason, the most invoked mechanism of action involves the contact between the cell and the charged surface, as first established by Isquith in 1972 [77]. In this respect, Tiller introduced the “*Polymeric Spacer Effect*” mechanism based on the presence of a spacer between the surface and the biocidal function, long enough to penetrate the OM and kill bacteria. For instance, long spacers of almost 19 nm (160,000 g mol^−1^) between the surface and the bactericidal function have been found to be crucial for penetrating the CW of gram-positive bacteria [41,78,79]. Similarly, a study on woven textile fabrics with covalently attached alkylated polyethyleneimines (PEIs) showed that the antibacterial activity depended on the length and thus on the molecular weight of PEIs and N-alkylation [80]. In fact, high-molecular-weight chains (750,000 Da, with a length of ca. 6 mm) rendered the textile highly bactericidal because of their ability to perforate the whole cell membrane of *S. aureus*; on the contrary, shorter chains (0.02–0.007 mm in length with average molecular weights spanning from 2000 to 800 Da) were ineffective as they were unable to penetrate and damage the bacteria. Nevertheless, surfaces bearing chains shorter than 800 Da could still exhibit antimicrobial activity depending on the nature of the polymer. In fact, surfaces grafted with fatty alkyl chains attached through quaternary amine anchors proved some bactericidal effect [77,81], which occurred according to the so-called “hole-poking” mechanism [78,82,83,84,85].

Indeed, the hypothesis based on the need for a polymeric spacer long enough to penetrate the CW or the OM of bacteria to make surfaces work as antimicrobial has been questioned in a few studies. In 2008, Matyjaszewski and coworkers [19] found that the biocidal activity of a glass surface grafted with quaternized poly(2-(dimethylamino)ethyl methacrylate) was not influenced by the length of polymer chains but rather by the surface charge density (*vide supra*). Similarly, other studies did not correlate the antimicrobial activity of polymeric surfaces to the extension of the polymer chains, as the antimicrobial surfaces described did not contain a polymeric spacer [86,87,88].

In this *scenario*, an alternative mechanism of contact killing was proposed by Kugler, who hypothesized the removal of the divalent stabilizing cations Mg^2+^ and Ca^2+^ from the OM of bacteria when they enter in contact with positively charged surfaces, the so-called “*Ion-Exchange mechanism*” [18]. Nevertheless, cell lysis, deriving from disruption of OM, is probably still working, as evidenced by the presence of cell constituents in a solution when coated materials [10,21,89] or polymer microbeads [90] containing QACs were mixed with a suspension of bacteria. For optimal biocidal efficiency of cationic surfaces, a minimum surface charge density is required [22,57,91], either to foster adhesion of bacterial cells on the surfaces or to cause leaching of Ca^2+^ and Mg^2+^.

**Figure 4:**
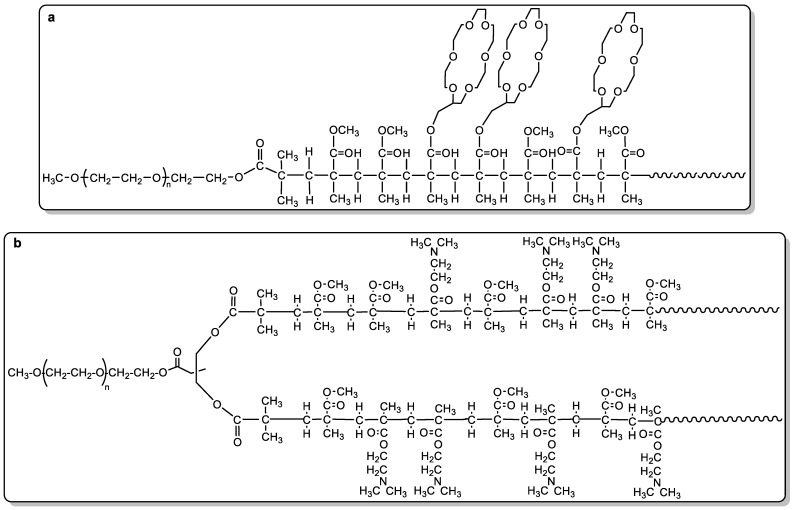
Copolymers with crown ether pedants and no biocidal functions [92] (**a**) and mPEG-(MMA-ran-DMAEMA)_2_ structures employed in antimicrobial mechanism of action studies ([54,95] (**b**).

To demonstrate that a key component of the “*Ion-Exchange mechanism*” was the loss of stabilizing ions, copolymers containing crown ether pedants and no biocidal functions were synthesized to introduce sequestering alkali-earth ions (Figure 4a); then, films produced from such copolymers were introduced in bacterial suspensions. The surfaces exhibited biocidal activity against *E. coli* [92], indirectly confirming the presence of a mechanism of action that starts by leaching or complexing the envelope-stabilizing cations. The complexing capacity of copolymers towards such alkali-earth cations was also proved by immersing a film in a solution containing Ca^2+^ and Mg^2+^ ions that were partially sequestered by the copolymer.

Although in the “*Ion-Exchange mechanism*” the effect of a destabilizing action on cellular membranes was recognized, experimental evidence of the specific cellular response was investigated in a study focused on the recognition of the possible regulators mediating the response to the damaging action. The study also permitted understanding of whether the antimicrobial action was a “passive” event, meaning it was only generated by polymer properties, or required a cellular response as induction of a death signal. To this end, the authors used a dual strategy, the first part consisting in the analysis of the cell damage caused by contact with the polymer surface, while the second part was based on the evaluation of the cell response at a genetic level. *E. coli* was used as the cell model, while the polymer surface was made of the monomethyl ether poly(ethylene glycol) (mPEG) covalently linked to two gradient-copolymeric chains based on MMA and DMAEMA (mPEG-(MMA-ran-DMAEMA)_2_, Figure 4b) [93,94,95] containing 34% in mol of non-quaternized DMAEMA, with the latter able to generate a charged surface by protonation when in contact with water. It had already been demonstrated that such a surface was active towards several gram-negative cells as well as the gram-positive *S. aureus* [54,55]. Interestingly, the gene expression study revealed that some key genes in the synthesis and maintenance of the OM structure along with some regulators of cellular response to oxidative stress were more expressed in bacteria exposed to a polymer surface [95]. On the other hand, the lithic effect on protoplasts of *E. coli* demonstrated that the polymer surface was able to act on the structure of cytoplasmatic membranes, while experiments on calcein leakage from unilamellar vesicles at different phospholipid compositions indicated that such action was present also in the absence of functionally active cells. All together, these results indicated that, once in contact with a positively charged surface, the increasing permeability of the OM generated by contact with the surface triggers the over-expression of specific genes in an attempt to counteract the membrane damage.

Another mechanism alternative to the “*Polymeric Spacer Effect*” was proposed by Tiller and Bieser in 2011. The so-called “*Phospholipid sponge effect*” is based on the adhesion of negatively charged phospholipids of the cell membrane onto the bactericidal, positively charged surface [96]. To prove this mechanism, the authors synthesized hydrophobic cellulose derivatives with a variety of QAC functions and hydrophobic neutral substituents. In detail, the biocidal surfaces active against the gram-positive *S. aureus* were made of N-alkyl-N,N-dimethyldeoxyammonium celluloses, obtained by converting tosyl celluloses with different degrees of substitution (DSs) [97] with the N,N-dimethyldodecyl-ammonium (DDA) group as the biocidal function and the N,N-dimethylbutylammonium (DBA) group, the latter considered non-biocidal by the authors. However, they found that some of the DBA containing celluloses were antimicrobially active against *S. aureus* by contact but, in contrast with the DDA derivatives, a certain number of tosylate groups must be present in this case, indicating that the tosylate hydrophobic functions support the antimicrobial action. The authors rationalized the experimental data by evoking a sort of balance between the charged and hydrophobic groups. Indeed, the biocidal DDA groups contain a hydrophobic tail, while DBA functions must be associated with hydrophobic groups even if distant from the cation. They suggested that this action was driven by the attraction between the negatively charged phospholipids of the bacteria and the surface, in stark contrast with the “*Polymeric Spacer Effect*” mechanism. However, the existence of both the mechanisms was proposed, along with a strategy indicating which one occurred in the specific cases. Indeed, while coatings with polymeric spacers between the biocidal group and the surface cannot be deactivated by chemical treatments, QAC-based antimicrobial coatings without polymeric spacers can be deactivated by treating the materials with an anionic surfactant (sodium dodecylsulfate, SDS), or with negatively charged phospholipids.

A shape-adaptive, contact-killing coating was prepared by Asri et al. by tethering QACs onto hyperbranched polyurea supported on a silicon substrate to enhance the contact area between bacteria and coatings [98]. The surface was able to kill adhering bacteria by partially enveloping them, and the “*Phospholipid sponge effect*” was proposed as the mechanism of action. As support for the mechanism, the authors mentioned Gottenbos [86], who in 2002 had already reported that the strength of bacterial adhesion to a surface may be a determining factor for their growth, and so-called “*stress-induced de-activation*” for bacteria strongly adhering to positively charged surfaces, a term used for the first time by Liu in [99] to indicate a reduced resistance of bacteria towards antimicrobials. The shape-adaptive hyperbranched Si-HB-PEI^+^ coating, due to the positively charged surface, exerted strong enough electrostatic forces to cause the removal of anionic lipids of the membrane through the outermost surface of the adhering cells of *Staphylococcus epidermidis*. Apparently, the Staphylococcal adhesion forces on the Si-HB-PEI^+^ coating exceeded the known forces by a factor of ca. six [100,101,102], justifying the electrostatic attraction of the anionic lipids of the membrane towards the positively charged surface, that induces localized membrane damage and causes bacterial death [103].

**Figure 5:**
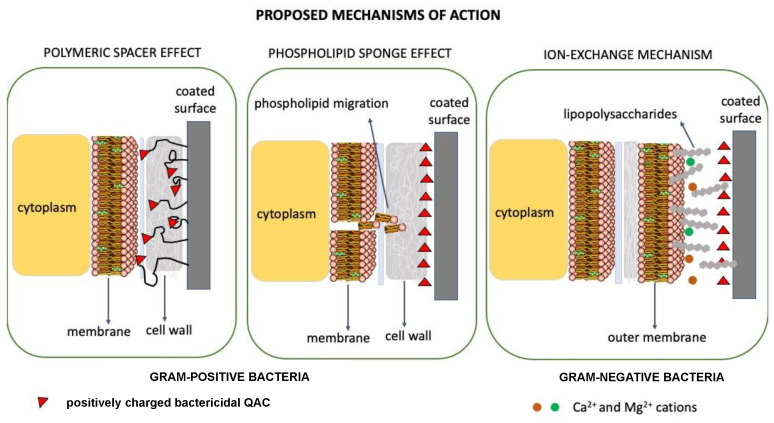
Representation of the antimicrobial mechanisms of action discussed in this review.

Overall, the last two mechanisms proposed both invoked the adhesion of cells to surfaces containing QACs as a key step for biocidal action. The “*Ion-Exchange mechanism*” considers the adhesion of bacteria as a consequence of the removal of the stabilizing mobile Ca^2+^ and Mg^2+^ cations by the positively charged surface and the following permeabilization of membranes that causes bacterial death; in the “*Phospholipid sponge effect*”, the strong electronic field generated by the positively charged surface is responsible for the surface adhesion by the negatively charged bacteria followed by electrostatic extraction of phospholipids from cell membranes (Figure 5).

A contribution to the comprehension of the antimicrobial action of charged surfaces comes from computational studies. A Monte Carlo simulation and coarse-grained models were used to evaluate how space restriction and absorption would influence the ionization degree, the conformation, and the counterion condensation of confined weak polyelectrolytes [104,105]. The authors connected their results to the fields of antimicrobial polymers and, in particular, to the antimicrobial activity of non-quaternized DMAEMA-based species [54,55,106,107]. The rationalization of the antimicrobial properties of such a species derives from the formation of strong hydrogen bonds between protonated and unprotonated amino groups of DMAEMA, (CH_3_)_2_NH^+^---N(CH_3_)_2_, enhancing the positive charge of the surface [108]. It was also found that the negative charge of the bacteria wall can contribute to increasing the positive charge up to a factor of 10 [109], while it was depressed when the polymer was confined within a vesicle. The suppression of the positive charge may have an impact on the number of negatively charged species on the wall surface migrating towards the polymer to compensate its charge, and hence on the mechanical resistance of the wall and on its tendency to rupture. Data obtained from the 1D confinement suggested that the ionization induced by the wall charge may be sufficient to bind DMAEMA oligomers and induce bacterial death. The distribution and density of charges on the surfaces and in a solution, depending on chemical structure, composition, and hydrophobicity [110,111,112], were also approached with simulation methods to better understand both the bacterial interaction with charged surfaces and the amount of the surface available for the bacteria [113].

Very recently, films made of non-quaternized mPEG-P(MMA-*ran*-DMAEMA)_2_ copolymers were produced by different techniques such as spin-coating, drop-casting, and casting deposition, obtaining ultrathin (16 ± 3 nm) and thick films (~400 mm) [114]. Their morphology was studied to deeply understand the correlation with the biocidal activity previously reported for thick films [54]. Drop-casted ultrathin and thick films were found to be morphologically flat, while spin-coated ultrathin films had a morphology dependent on the substrate. The hardness of the surface was investigated by AM-AMF measuring the Young modulus and the viscoelastic behavior of the films [115,116] in both air and water, environments similar to those used in antimicrobial tests. When in water, the swelling made the films softer at the surface. In particular, the large reduction in the Young modulus of the thick samples was suggested as a factor able to promote a conformal contact between the bacteria and the film, also indicating the role of mechanical properties of surfaces in antimicrobial activity.

## 4. Conclusions and Future Perspectives

It is widely reported that contaminated surfaces play a determining role in bacterial infection processes; thus, the rational design of antimicrobial surfaces represents the key to reducing or, in some cases, avoiding the spread of microorganisms. Although so far scientists have made many efforts to understand the correlation between surfaces and the microorganisms’ response to their exposure, further attempts to improve the effectiveness or the selectivity of antimicrobial polymer surfaces are still needed. QACs are among the most powerful antimicrobial functions and hence are used in many industrial products. When immobilized on polymer surfaces, their biocidal activity is preserved. Due to a generally low hemolytic activity, polymer surfaces containing QACs have potential applications in widely varying fields, such as surgical, medical implant, or wound dressing applications [117], and more generally, in industrial equipment [118,119], water purification systems [120], and food packaging [74,121].

In this review, we focused on the synthesis of polymer coatings on pre-existing surfaces as well as on water-insoluble, inherently antimicrobial polymers forming surfaces. In both cases, advanced coating and polymerization techniques along with relevant examples have been described. Although the biocidal activity of such surfaces occurs by contact with bacterial cells, the mechanism is still under debate; hence, we discussed those mechanisms widely accepted by the scientific community.

More generally, advanced synthetic approaches for obtaining antimicrobial polymer surfaces and a deep comprehension of their mechanism of action represent two sides of the same coin, hence they are both crucial for the harmonious development of surfaces with more effective antibacterial activity. To this end, a significant improvement in the field would derive from a strategy similar to that adopted in nanotherapeutics for infectious diseases [122], namely to consider the bacterial structures to properly develop targeted polymers that, increasing the surface affinity towards specific cells, would also improve adhesion to and selectivity towards bacteria [123]. Another important aspect of the development of antimicrobial polymer surfaces is related to the recent SARS-CoV-2 pandemic, which dramatically increased the demand for antimicrobial treatments of contaminated surfaces especially in healthcare settings. In this respect, amine-based dendrimers and polyethyleneimine can be effective as potential inhibitors and for immunization against coronaviruses [124,125], paving the way to the development of combined antiviral and antibacterial polymer surfaces.

Finally, a meaningful aspect that could involve the future development of antimicrobial polymer surfaces is the worldwide increasing attention towards green economy. The expanding availability of molecules from renewable sources/biomass is inspiring the synthesis of novel and sustainable polymer-based materials with the aim of replacing those deriving from fossils. Natural molecules are, however, often multifunctional, and the additional substituents, other than the polymerizable ones, may be antimicrobial themselves or, in principle, be advantageously used for post-functionalization to introduce QACs and produce added-value, bio-based biocidal polymers [126]. In this respect, the development of increasingly advanced and precise techniques of polymerization [127] aiming at preserving functional groups of multifunctional natural molecules, as well as the development of advanced methods of surface functionalization, will allow for the rational design and synthesis of novel and more efficient antibacterial surfaces starting from the knowledge of the biocidal behavior and mechanism of action of the already reported polymer surfaces containing QACs.

In conclusion, we are convinced that future strategies for the development of advanced antimicrobial polymer surfaces for modern human healthcare should integrate the material’s design, preferably starting from bio-based molecules, and microorganisms’ biology for the preparation of targeted, selective, and more effective surfaces or, when needed, with combined antiviral and antibacterial activity.

## Figures and Tables

**Table 1:** Summary of the water-insoluble antimicrobial polymers discussed in Section 2.2.

Type of Polymer	Tested Species	References
poly-(Quaternary amine methacrylate)-*co*-(2-hydroxyethyl methacrylate)	*E. coli* *S. aureus*	[43,44,45]
DMAEMA	*E. coli*	[46]
polyether ether keton	*A. niger*, *A. pullulans*, *C. globosum*, *G. virens*, *P. pinophilum*	[47,48,49]
polyethylene terephthalate	*E. coli*	[50,51]
poly-(vinylbenzyl chloride)-*co*-(*m*ethylmethacrylate)/(2-chloroethylvinyl ether)	*C. albicans* SC5314, *A. flavus*, *F. oxysporum*; *B. subtilis*, *E. coli*, *S. aureus*	[52]
poly-(ε-caprolactone)-*co*-(α-cloro-ε-caprolactone	*E. coli*	[53]
(PEG)-MMA-*ran*-DMAEMA	*E. coli*, *S. aureus*, *Pseudomonas* sp., *Dechlorosoma* sp.	[54,55]
(methacrylamide) -poly-(2-methyloxazoline)- (N-dodecyl-N,N-dimethylxylylammonium bromide)	*S. mutans*	[56]
poly-(3-(Methacrylamidomethyl)-pyridine)-*ran*-(N-isopropylacrylamide)	*E. coli, S. Aureus*	[57]
poly-(4-vinyl pyridine)-*ran*-(HEMA)/(PEGMA)	*E. coli*	[58]
poly-(vinylpyridium halide)-based resins	various bacteria and viruses	[59,60,61,62]
ionene polymers	*P. aeruginosa*, *K. pneumoniae*, *E. feacalis*, *M. luteus*, *B. subtilis* var. *niger*, *C. albicans*, *P. digitatum, A. niger*	[63,64,65]
modified polysaccharides	various bacteria	[66]
